# Kinin B_1_ Receptor Agonist Enhances Blood-Brain Barrier Permeability in Healthy and Glioblastoma Environments

**DOI:** 10.3390/ph18040591

**Published:** 2025-04-18

**Authors:** Carolina Batista, João Victor Roza Cruz, Michele Siqueira, João Bosco Pesquero, Joice Stipursky, Fabio de Almeida Mendes

**Affiliations:** 1 Instituto de Ciências Biomédicas, Universidade Federal do Rio de Janeiro, Rio de Janeiro 21941-902, RJ, Brazil; carolinabatista@icb.ufrj.br (C.B.); joao.cruz@ensino.inca.gov.br (J.V.R.C.); michelessqr@gmail.com (M.S.); joice@icb.ufrj.br (J.S.); 2 Departamento de Biofísica, Universidade Federal de São Paulo, São Paulo 04039-032, SP, Brazil; jbpesquero@unifesp.br

**Keywords:** kinin B_1_ receptor, des-Arg^9^-bradykinin, blood-brain barrier, endothelial cells, glioblastoma

## Abstract

**Background/Objectives**: The low permeability of the blood-brain barrier (BBB) represents a significant challenge to effective systemic chemotherapy for primary and metastatic brain cancers. Kinin receptors play a crucial role in modulating BBB permeability, and their agonist analogs have been explored in preclinical animal models to enhance drug delivery to the brain. In this study, we investigated whether des-Arg^9^-bradykinin (DBK), a physiological agonist of kinin B_1_ receptor (B1R), acts as a brain drug delivery adjuvant by promoting the transient opening of the BBB. **Methods**: Human brain microvascular endothelial cells (HBMECs) were treated with DBK in the culture medium and in conditioned media from glioblastoma cell lines, namely T98G (CMT98G) and U87MG (CMU87). Immunofluorescence, RT-qPCR, in-cell Western assay, and proximity ligation assay (PLA) were performed to analyze BBB components, kinin receptors and TLR4, a receptor associated with the kinin pathway and inflammation. The effect of DBK on enhancing paracellular molecule transport was evaluated using Evans blue dye (EB) quantification in a cell culture insert assay and in an in vivo model, where mice with and without brain tumors were treated with DBK. To assess the functional impact of the transient BBB opening induced by DBK, the chemotherapeutic drug doxorubicin (DOX) was administered. **Results**: Treatment with DBK facilitates the presence of EB in the brain parenchyma by transiently disrupting the BBB, as further evidenced by the increased paracellular passage of the dye in an in vitro assay. B1R activation by DBK induces transient BBB opening lasting less than 48 h, enhancing the bioavailability of the DOX within the brain parenchyma and glioma tumor mass. The interaction between B1R and TLR4 is disrupted by the secreted factors released by glioblastoma cells, as conditioned media from T98G and U87 reduce TLR4 staining in endothelial cells without affecting B1R expression. **Conclusions**: These results further support the potential of B1R activation as a strategy to enhance targeted drug delivery to the brain.

## 1. Introduction

Inflammation is the immune system’s response to harmful stimuli, such as pathogens and damaged cells, with the goal of eliminating these threats and initiating the healing process [1]. Among the various mediators of inflammation, kinins play a significant role [1,2], acting specifically on blood vessels [3,4].

Kinins are peptides released during tissue injury and are rapidly inactivated by enzymes present in tissues and plasma [2,3,4,5,6,7]. This process generates biologically active metabolites and inactive peptides [2,4,5,6,7]. Their biological effects are mediated through the activation of two receptors, B1R and B2R [4,8,9]. Bradykinin (BK) (Arg-Pro-Pro-Gly-Phe-Ser-Pro-Phe-Arg) and Lys-bradykinin (Lys-BK) (Lys-Arg-Pro-Pro-Gly-Phe-Ser-Pro-Phe-Arg) exhibit high affinity for B2R, while the active metabolites DBK (Arg-Pro-Pro-Gly-Phe-Ser-Pro-Phe) and Lys-des-Arg^9^-bradykinin (Lys-DBK) (Lys-Arg-Pro-Pro-Gly-Phe-Ser-Pro-Phe) preferentially bind to B1R [4,5,10,11,12]. B2R receptors are constitutively expressed in most tissues, whereas B1R receptors are either absent or weakly expressed under normal conditions. However, in inflammatory environments, B1R expression can be upregulated by pro-inflammatory mediators [5,9,10,13,14,15,16]. Additionally, pro-inflammatory and oxidative stress conditions in the tumor microenvironment promote the overexpression of kinin receptors [17].

Cancer remains one of the leading causes of disease-related mortality worldwide, exerting a profound impact on society [18]. Glioblastoma, the most common and aggressive type of brain tumor, originates from astrocytic cells [19,20]. Within the glioblastoma microenvironment, aberrant signaling associated with tissue damage can influence the phenotype and activation status of tumor cells [21,22,23]. These cells, in turn, regulate the functions of both cellular and non-cellular components of the tumor microenvironment, modulating metabolic demands, immune surveillance, survival, invasion, and the maintenance of glioma stem cells; additionally, they promote microvascular proliferation and/or necrosis [22,23,24,25].

The role of kinins and their receptors in glioblastoma has not yet been fully elucidated. However, the existing literature suggests that the expression of these receptors in glioma cells is associated with tumor grading and developmental stage [9,26,27,28]. The release of BK and its active metabolites, along with their interactions with B1R or B2R, contribute to pathological conditions, often characterized by increased vascular permeability [2,8].

The BBB is a selective cellular barrier that separates blood components from the brain’s microenvironment, regulating the entry and exit of ions, nutrients, macromolecules, and metabolites; this protects the central nervous system (CNS) from neurotoxic substances while ensuring proper brain nourishment [20,29,30,31,32]. The BBB is composed of a monolayer of brain endothelial cells connected by tight junctions and adherent junctions, with transmembrane proteins localized at cell-to-cell contacts; intracellular junctional scaffolding proteins interact with neighboring cells, such as astrocytes, pericytes, and perivascular macrophages [20,29,30,31,33,34,35,36]. Since tight junction proteins, such as zonula occludens-1 (ZO-1) and claudin-5, severely restrict the entry of hydrophilic drugs and limit the penetration of larger molecules, such as peptides, strategies for drug delivery to the CNS must account for these barriers [29]. Over the past few decades, several strategies have been explored to enhance the delivery of therapeutic agents to brain tumors; however, they have yet to significantly changes tumor therapies, which, in the case of glioblastoma, typically involve surgical resection followed by adjuvant radiotherapy in combination with the chemotherapy agent temozolomide (TMZ) [30,34,36,37,38].

It is evident that therapeutic permeabilization of the BBB should be as brief as possible to minimize potential side effects [29,39]. Approaches using intracarotid drug infusions have shown some success in enhancing drug delivery to brain tumors, particularly with the use of inflammatory mediator analogs of BK [29,39,40,41,42]. However, clinical trials have highlighted the need for further refinement of this approach [29,30,33,36,39,41,43,44].

Pharmacological analogs of B1R and B2R agonists have improved the bioavailability of carboplatin [43] and DOX [45] in glioblastoma tumor masses, enhancing survival in animal models. One way to enhance vascular sensitivity to DBK is through the interaction of lipopolysaccharide (LPS) with TLR4, which induces the synthesis of pro-inflammatory cytokines [46,47]. LPS, a component of the outer membrane of Gram-negative bacteria, is an agonist of the TLR4 and can modify gene expression in various cell types; it has also been identified as a potential anti-tumor agent for glioblastoma treatment [8,10,11,13,37,48]. Despite its potent pro-inflammatory properties, no treatments using LPS-like agents have been approved by regulatory agencies [37,49]. In this study, the strategy to transiently open the BBB involves enhancing the activity of B1R through its physiological agonist, DBK.

The relationship between kinin receptors and Toll-like receptors has been described in various cell types, where co-stimulation of Toll-like receptors or LPS treatment leads to the transcription of *B1R* and *B2R* via the nuclear factor kappa B (NF-κB) pathway [50,51]. Previous work from our group has further demonstrated that TLR4 activation by LPS enhances B1R activity, suggesting that this interaction may have therapeutic potential, such as facilitating BBB permeability, a major challenge in brain tumor therapies [52,53]. Like LPS, DBK also promotes a physical proximity of the receptors, bringing them to the perinuclear and nuclear regions [53].

Considering the relationship between B1R and TLR4 and the previously described effects of B1R and B2R agonist analogs on the BBB, we investigated whether B1R activation promotes transient BBB opening, which could facilitate drug bioavailability to the brain parenchyma, both with and without a tumor mass.

## 2. Results

### 2.1. Effect of T98G- and U87MG-Conditioned Media on Endothelial Cells

**Figure 1:**
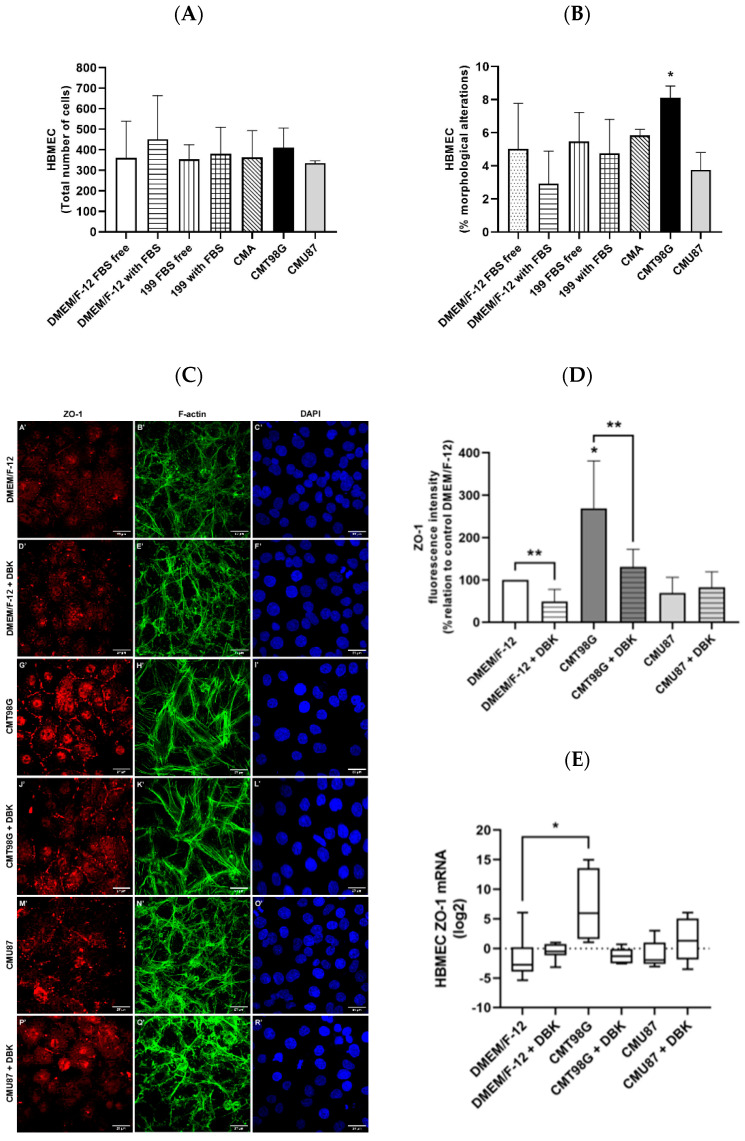
Effect of conditioned media from U87MG and T98G on HBMECs, with and without DBK. (**A**) None of the conditions resulted in a change in total cell count. (**B**) After 24 h of exposure, the percentage of HBMECs with spread-out morphology was counted. HBMECs treated with CMT98G exhibited an increased percentage of cells with morphological alterations, compared to cells exposed to DMEM/F-12 with FBS or U87-conditioned medium (CMU87). (**C**) Immunocytochemistry images (**A’**–**R’**) showing ZO-1, F-actin, and nuclear staining (DAPI) in control conditions (DMEM/F-12 with FBS) and in glioblastoma-conditioned medium (CMT98G and CMU87), with or without 1 µM DBK. (**D**) Quantification of ZO-1 immunofluorescence intensity reveals a significant increase in HBMECs exposed to CMT98G. The addition of DBK to both control and CMT98G conditions reduces ZO-1 fluorescence intensity. (**E**) HBMECs exposed to CMT98G show increased ZO-1mRNA levels, which return to control levels upon the addition of 1 µM DBK. (**D**) * *p* < 0.05 vs. DMEM/F-12, DMEM/F-12 + DBK, CMU87, and CMU87 + DBK; ** *p* < 0.05 DMEM/F-12 vs. DMEM/F-12 + DBK, CMT98G vs. CMT98G + DBK, unpaired *t*-test. (**E**) * *p* < 0.05 MCT98G vs. DMEM/F-12 + DBK. (**B**) * *p* < 0.05 vs. DMEM/F-12 with serum and CMU87. (Kruskal–Wallis test and ANOVA (Holm–Sidak test)). Scale bar: 27 µm. Number of experiments: (**A**,**B**) 5, (**C**–**E**) 7.

The initial analysis focused on the behavior of brain endothelial cells when exposed to conditioned media derived from glioblastoma cell lines. Phase-contrast imaging revealed no reduction in the total number of cells across the tested conditions (Figure 1A). However, cultures treated for 24 h with conditioned media from glioblastoma cell line T98G (CMT98G) exhibited an increase in cells with altered, spread-out morphology within the monolayer (Figure 1B) [54,55]. This morphological change was significantly different from that observed in cells exposed to cell culture media DMEM/F-12 with fetal bovine serum (FBS) and conditioned media from glioblastoma cell line U87 (CMU87), where the cells exhibited a cobblestone morphology, which is a typical characteristic of endothelial cells, and their morphology was maintained [56,57]. To further investigate these changes, the cultures were fixed and stained for ZO-1 and F-actin, a cytoskeletal component. Using 199 medium supplemented with FBS as a control, the analysis of ZO-1 staining fluorescence intensity reveals a decrease in fluorescence in cells maintained for 24 h in DMEM/F-12 without FBS. Interestingly, when comparing the ZO-1 fluorescence intensity of cells exposed to CMT98G and CMU87 with those exposed to DMEM/F-12 with FBS, an increase in fluorescence intensity was observed in HBMECs treated with CMT98G (Figure 1C,D). This increase could indicate either an upregulation of ZO-1 protein levels within the barrier structure or a compensatory response to restore a disorganized barrier. After this, HBMECs were incubated with 1 µM DBK to analyze whether modulation of the B1R could affect endothelial cells and ZO-1. The immunofluorescence intensity analysis of the ZO-1 (Figure 1C) showed a decrease in fluorescence intensity when DBK is administered alongside CMT98G (Figure 1C(J’)). Similarly, cells incubated with DBK in DMEM/F-12 also exhibited lower fluorescence intensity compared to DMEM/F-12 control (Figure 1C(A’,D’)). In contrast, no variation in ZO-1 fluorescence intensity was observed when DBK was added to CMU87 (Figure 1C(M’,P’)). RT-qPCR data indicated that HBMECs incubated with CMT98G exhibited increased mRNA levels of ZO-1, which returned to control levels upon the addition of DBK to the medium (Figure 1E). These results align with the fluorescence intensity data (Figure 1D) and suggest that B1R activity may modulate ZO-1 expression in a glioblastoma cell line-dependent manner. Similar to ZO-1, claudin-5 mRNA levels increase in HBMECs treated for 24 h with MCT98G but appear to decrease when DBK is incorporated into the conditioned medium (Figure S1). There is no change in the mRNA levels of this BBB component in HBMECs exposed to MCU87, regardless of the presence or absence of 1 µM DBK.

### 2.2. B1R and TLR4 Analysis in BBB Endothelial Cells Exposed to CMU87 and CMT98G in the Presence or Absence of DBK

**Figure 2:**
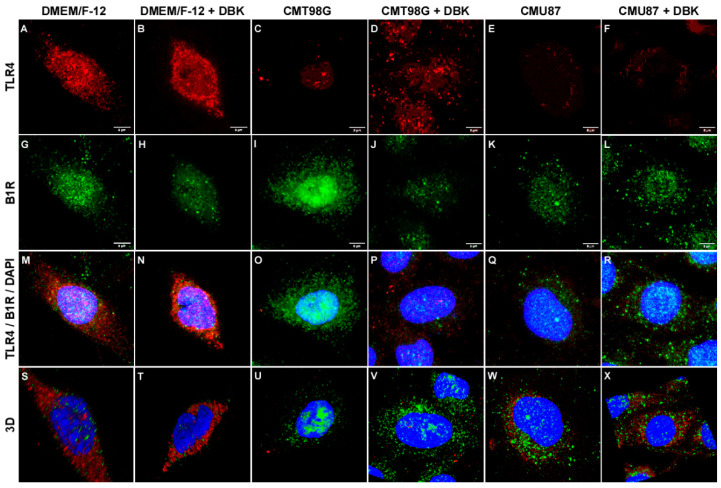
B1R and TLR4 labeling patterns in endothelial cells treated with CMT98G and CMU87, with or without DBK. HBMECs cultured in DMEM/F-12 medium (control) (**A**,**G**,**M**,**S**) and treated with DBK (**B**,**H**,**N**,**T**) exhibit diffuse TLR4 (**A**) and B1R (**G**) staining throughout the cell, which becomes concentrated in the perinuclear region upon DBK treatment (**B**,**H**). HBMECs exposed to CMT98G show reduced TLR4 labeling both in the absence (**C**) and presence (**D**) of DBK, while B1R labeling is prominent in the perinuclear region in cells exposed to CMT98G (**I**) but decreases upon DBK treatment (**J**). In cells exposed to CMU87, TLR4 labeling is reduced without (**E**) and with DBK (**F**), whereas B1R labeling remains intense in the perinuclear region regardless of the presence (**K**) or absence (**L**) of DBK. (**M**–**R**) Combined images of TLR4, B1R, and DAPI staining. (**S**–**X**) 3D reconstruction of triple-labeled images. Scale bar: 6 µm Number of experiments = 10.

As previously described by our group [53], HBMECs exposed to DMEM/F-12 medium (control condition) exhibit diffuse labeling of B1R and TLR4 (Figure 2A,G), with distribution throughout the cell. However, upon treatment with DBK, the staining becomes concentrated in the perinuclear region (Figure 2B,H).

In HBMECs exposed to conditioned media from glioblastoma cell lines (MCT98G or MCU87), a decrease in TLR4 staining was observed, both in the absence (Figure 2C,E) and presence of DBK (Figure 2D,F). B1R receptor staining was concentrated in the perinuclear region of HBMECs exposed to CMT98G (Figure 2I); however, this concentration decreased upon the addition of DBK (Figure 2J). In cells exposed to CMU87, B1R staining is more intense in the perinuclear region, both in the absence (Figure 2K) and presence (Figure 2L) of 1 µM DBK in the medium.

**Figure 3:**
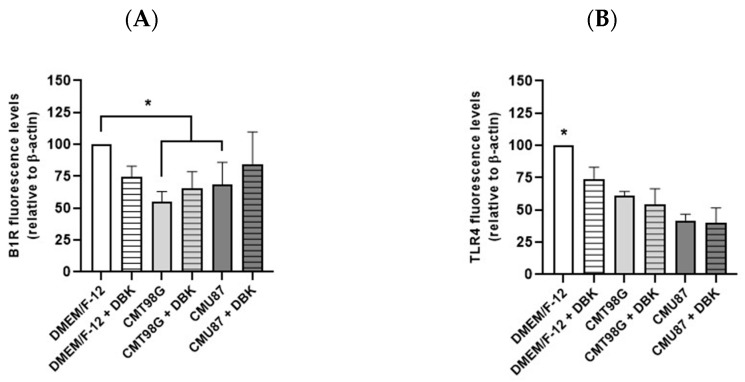

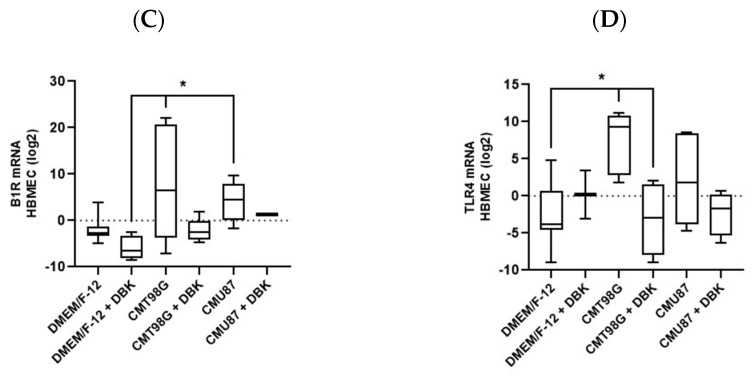
Relative quantification of B1R and TLR4. (**A**,**B**) In-cell Western assay relative quantification of B1R and TLR4 proteins. B1R protein did not vary under the DBK conditions but decreased significantly in cells exposed to CMT98G and CMU87 without DBK (**A**). TLR4 expression decreased significantly in HBMECs exposed to CMT98G e CMU87 (**B**). (**C**,**D**) Relative mRNA expression levels of B1R and TLR4 receptors in endothelial cells incubated for 24 h with CMT98G or CMU87, with or without DBK (**C**). B1R mRNA increased in cells exposed to CMT98G and CMU87 compared to the DMEM/F-12 + DBK. CMU87 compared to DMEM/F-12 + DBK (**C**). TLR4 mRNA expression increased in cells treated with CMT98G and CMT98G + DBK compared to the DMEM/F-12 control (**D**). (**A**) * *p* < 0.05 DMEM/F-12 vs. CMT98G, CMT98G + DBK, CMU87. (**B**) * *p* < 0.05 DMEM/F-12 vs. CMT98G, CMT98G + DBK, CMU87, CMU87 + DBK. (**C**) * *p* < 0.05 DMEM/F-12 + DBK vs. CMT98G and CMU87. (**D**) * *p* < 0.05 DMEM/F-12 vs. CMT98G and CMT98G + DBK (Dunnett’s test and Kruskal–Wallis test). Number of experiments: (**A**,**B**) 3 and (**C**,**D**) 9.

Relative quantification of B1R and TLR4 staining in control and treated HBMECs was performed using an in-cell Western assay (Figure 3A,B and Figure S2). No variation in the B1R protein expression was observed under DBK conditions compared to the control. However, a significant decrease was noted in cells exposed to CMT98G, both with or without DBK, and CMU87 without DBK (Figure 3A). TLR4 expression significantly decreased in HBMECs exposed to CMT98G e CMU87 (Figure 3B). The cell density of each well was stained with crystal violet. The comparison between immunofluorescence and in-cell Western assays suggests that, in CMT98G treated cells, the receptors are concentrated in the perinuclear region, a distribution that disappears upon peptide addition. This behavior is opposite to what is observed in the control condition and may be associated with the reduction in TLR4. Analysis of mRNA expression levels revealed an increase in B1R mRNA in cells treated with CMT98G and CMU87, with or without DBK, compared to DMEM/F-12 + DBK (Figure 3C). Additionally, TLR4 mRNA expression increased in cells treated with CMT98G and CMT98G + DBK (Figure 3D). The analysis of B2R expression indicated increased mRNA expression in HBMECs exposed to conditioned media and to MCT98G + DBK compared to the control condition (Figure S3). The transcription factor NF-κB, associated with TLR4 and B1R, showed no significant changes in mRNA expression in endothelial cells exposed to tumor microenvironments, with or without DBK.

**Figure 4:**
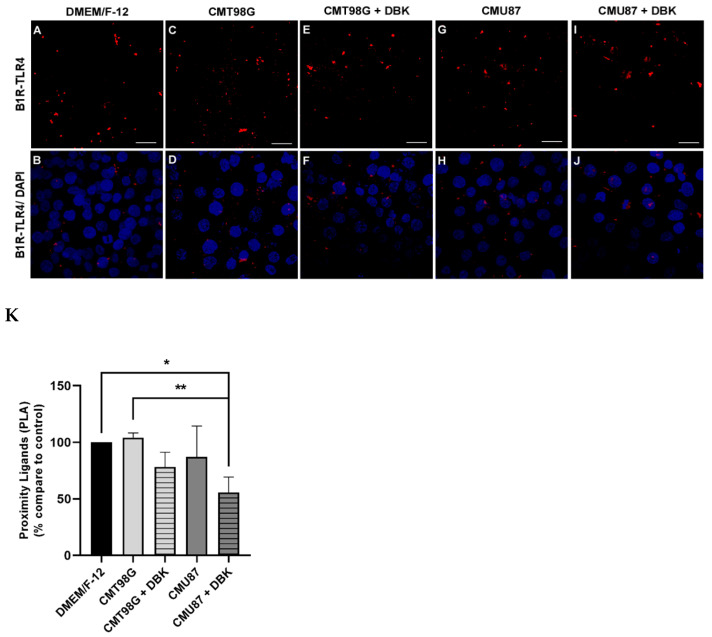
Reduction in proximity points between B1R and TLR4 in HBMECs incubated with CMT98G and CMU87 media enriched with DBK. The PLA for observing the proximity between B1R and TLR4 receptors in HBMEC cultures shows binding points under control conditions (DMEM/F-12 medium) (**A**,**B**), and after 24 h of incubation with CMT98G (**C**,**D**) and CMU87 (**G**,**H**), with or without 1 µM DBK (**E**,**F**,**I**,**J**). (**K**) Quantification of binding points reveals a significant reduction in the number of points in the CMU87 + DBK condition compared to DMEM/F-12 and CMT98G. (**K**) * *p* < 0.05 CMU87 + DBK vs. DMEM/F-12; ** *p* < 0.05 CMU87 + DBK vs. CMT98G (Tukey’s test). Scale bar: 27 µm. Number of experiments = 3.

A proximity ligation assay (PLA), can detect protein interactions in situ, within 40 nanometers, and precisely localize protein targets at a single-molecule resolution. As described by Batista et al. (2024) [53], it was observed whether there is any physical interaction between the B1R and TLR4 receptors or if the interaction occurs solely indirectly. The PLA showed an increased physical proximity between these receptors when DBK or LPS were present in the culture medium, indicating activation of these receptors [53]. In the present study, the PLA performed on HBMECs incubated for 24 h with CMT98G and CMU87 revealed that, compared to the control condition (DMEM/F-12), the conditioned media alone did not affect the proximity points (less than 40 nm) between B1R and TLR4 (Figure 4A–D,G,H). However, even with the addition of 1 µM DBK to the CMT98G and CMU87MG conditions, no changes were observed in the proximity points for CMT98G. In contrast, a reduction in these proximity points was seen in the CMU87 + DBK condition (Figure 4E,F,I,J), with a significant decrease compared to the control condition. (Figure 4K).

### 2.3. DBK Promotes Transient Opening of the BBB

**Figure 5:**
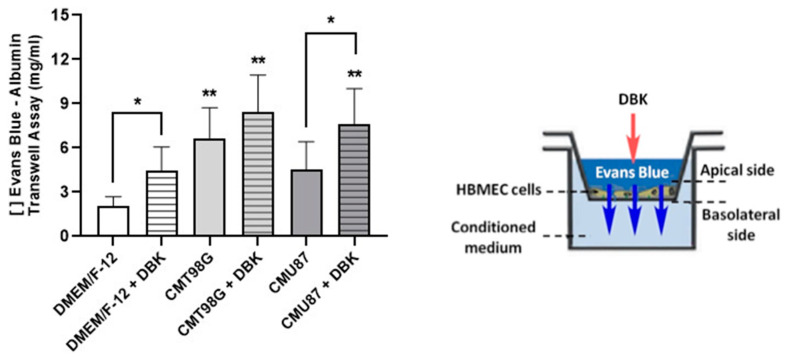
Paracellular transport of the Evans blue–BSA complex in an insert/transwell assay with HBMECs. The addition of 1 µM DBK to DMEM/F-12 medium facilitates dye transport from the apical to the basal region. CMT98G increased permeability even without DBK, while CMU87 required DBK for significant dye passage. * *p* < 0.05 DMEM/F-12 vs. DMEM/F-12 + DBK and CMU87 vs. CMU87 + DBK; ** *p* < 0.05 DMEM/F-12 vs. CMT98G, CMT98G + DBK, and CMU87 + DBK (Kruskal–Wallis test). Number of experiments = 6.

To assess whether DBK promotes paracellular transport, a transient permeability assay was performed using EB dye. The addition of 1 µM DBK in DMEM/F-12 medium facilitated the passage of the dye from the apical to the basal region (Figure 5). CMT98G in the basal chamber induced significantly higher dye passage even without DBK, an effect not observed with CMU87, where DBK was required for significant dye passage compared to the control.

**Figure 6:**
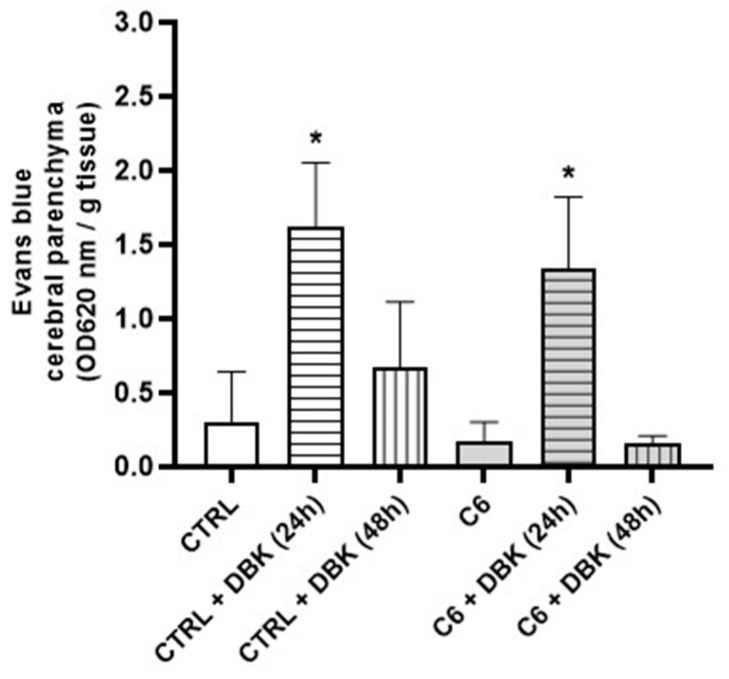
DBK induces a transient opening of the BBB for less than 48 h. Analysis of Evans blue dye in the cerebral parenchyma of animals, with or without C6 cell inoculation, shows the BBB opening 24 h after DBK injection and closure by 48 h. * *p* < 0.05 CTRL + DBK (24 h) and C6 + DBK (24 h) vs. CTRL, CTRL + DBK (48 h), C6, C6 + DBK (48 h) (Holm–Sidak test). Number of experiments = 5.

In vivo analysis of Evans blue dye in the brain parenchyma confirmed that the BBB opened 24 h after DBK injection in both control animals and those inoculated with C6 cells (Figure 6). By 48 h, dye levels in the parenchyma returned to baseline, confirming the transient nature of the opening. Variability in tumor size among animals likely contributed to statistical variations (Figure 6).

**Figure 7:**
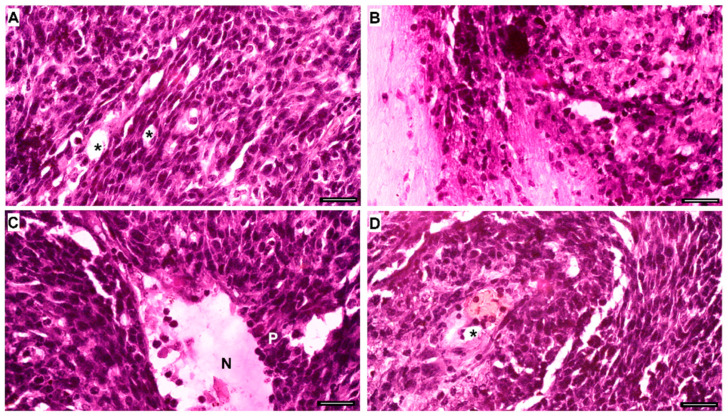
Histopathological characteristics of a C6 cell tumor. The C6 glioma is composed of a massive accumulation of C6 cells with typical features of glioblastoma multiforme, including high cellularity (**A**), regions of brain parenchyma invasion (**B**), and the presence of pseudopalisading necrotic foci (N and P in (**C**)). The cells exhibit nuclei ranging from round to oblong, with a fishbone-like growth pattern (**A**,**D**), as well as mitotic cells and microvessels (asterisks in **A**,**D**). Light microscopy, scale bar: 25 µm.

Histological sections of brain tissue (6–10 µm thick) from control mice and those inoculated with C6 cells under different treatments were stained with crystal violet to assess tumor presence. This analysis is crucial due to the significant variation in tumor size among animals, as C6 cells are of rat origin (Figure S4). Histopathological examination of tumors at the cellular level, performed on H&E-stained sections, revealed a massive accumulation of C6 cells displaying hallmark glioblastoma characteristics (Figure 7). These features include high cellularity, invasion of the brain parenchyma, and necrotic foci surrounded by pseudopalisade cells. Additionally, C6 gliomas exhibit diverse nuclear morphology (ranging from round to oblong), a fishbone-like growth pattern, mitotic figures, and microvascular proliferation [58,59,60,61].

### 2.4. DBK Effect on DOX Delivery to the Cerebral Parenchyma

**Figure 8:**
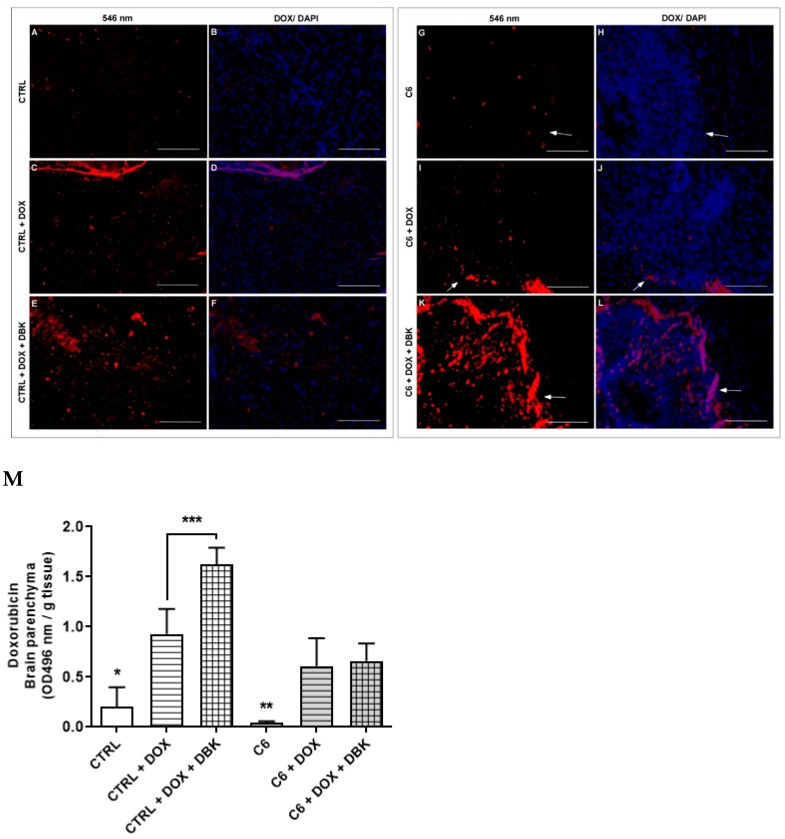
DBK enhances DOX bioavailability in the cerebral parenchyma and C6 tumor mass. In control mouse brain tissue, background fluorescence at 546 nm was detected (**A**,**B**). In mice receiving only DOX, minimal background fluorescence was observed (**C**,**D**). However, in animals pretreated with 1 µM DBK one hour before DOX administration and sacrificed 24 h later, DOX fluorescence was clearly evident (**E**,**F**). In brain tissue from C6 tumor-bearing mice (white arrows in the images indicate the peritumoral region), no specific fluorescence was detected at 546 nm in animals that did not receive DOX or DBK (**G**,**H**). In mice treated with DOX alone, fluorescence was present in the tumor area, predominantly in the peritumoral region (**I**,**J**). Notably, in animals pretreated with 1 µM DBK, an increased presence of DOX fluorescence was observed in the tumor tissue (**K**,**L**). (**M**) After DBK injection (24 h) and DOX administration (23 h), DOX was detected in both control and C6 tumor-bearing mice. In control animals without tumors, DOX levels were significantly higher when DBK was administered before chemotherapy. However, in tumor-bearing mice, no significant difference was observed between those pretreated with DBK and those receiving DOX alone. (**M**) * *p* < 0.05 CTRL and ** *p* < 0.05 C6 vs CTRL + DOX, CTRL + DOX + DBK, C6 + DOX, C6 + DOX + DBK; *** *p* < 0.05 CTRL + DOX vs CTRL + DOX + DBK(Holm–Sidak test). Scale bar: 150 µm. Number of animals per condition: (**A**–**F**) 5, (**G**–**L**) 3, and (**M**) 3.

To determine whether transient BBB opening induced by DBK facilitates the passage of a typically impermeable chemotherapeutic agent into the cerebral parenchyma, an in vivo assay was conducted using DOX in combination with DBK. Histological analysis of brain sections from control mice without C6 tumors sacrificed 24 h after DBK injection (23 h after DOX administration) confirmed the presence of DOX in the brain tissue (Figure 8), consistent with previous findings [45].

In these control animals, background fluorescence (546 nm emission) was observed following DBK and DOX administration (Figure 8A). In contrast, mice receiving only DOX exhibited detectable fluorescence in the parenchyma (Figure 8C), which became more intense when DBK was administered prior to DOX (Figure 8E). In the control condition of animals inoculated with C6 tumors, background fluorescence was observed at 546 nm emission (Figure 8G). Arrows in the figures highlight the peritumoral region, with the tumor area characterized by higher nuclear density (marked with DAPI), as corroborated by histological findings (Figure 7 and Figure S4). In brain sections from tumor-bearing mice, DOX was detected in both the tumor region and the peritumoral areas (arrows in Figure 8I,K). Notably, in animals pretreated with DBK, DOX was consistently more concentrated in the tumor region than in those receiving DOX alone.

DOX levels in the brains of control mice without C6 tumors were significantly higher when 1 µM DBK was administered (Figure 8M). However, in C6 tumor-bearing mice, no significant difference was observed between groups receiving DBK before DOX and those receiving DOX alone. This suggests that in the presence of the tumor, DBK does not further enhance DOX permeability across the BBB. Instead, the C6 tumor itself may contribute to increased ZO-1 expression, similar to the effect observed in endothelial cells exposed to CMT98G, but in a more organized and functional manner.

**Figure 9:**
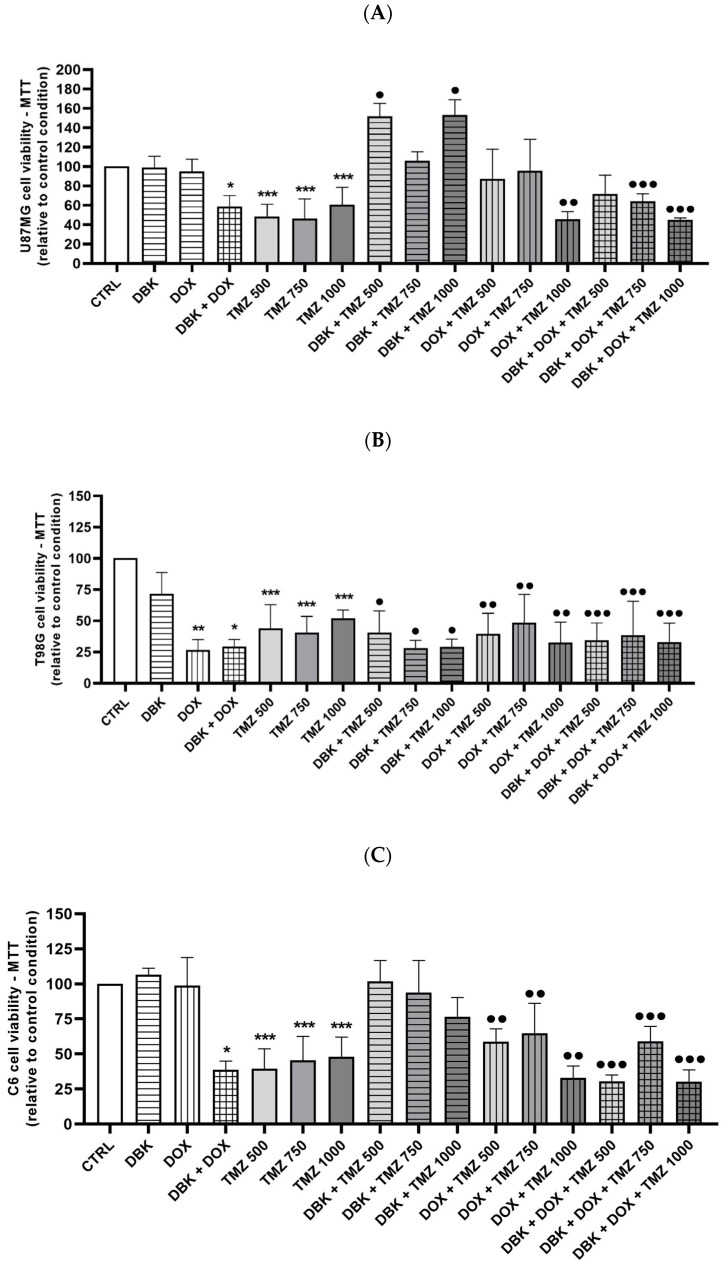
Cell viability assay in glioblastoma cell lines after DBK exposure. (**A**) In U87MG cells, DBK appears to enhance cell viability when combined with TMZ, potentially reducing its effectiveness. Additionally, combining TMZ with DOX or with DOX + DBK does not further reduce cell viability. (**B**) In T98G cells, treatment with 1 µM DBK reduces cell viability in all tested conditions. (**C**) In the murine C6 cell line, TMZ effectively reduces cell viability. DOX alone has little or no effect; but when combined with DBK, it significantly inhibits cell proliferation. Similar to U87MG cells, the combination of DOX with TMZ, as well as DOX + DBK + TMZ, does not provide additional reductions in proliferation. (**A**) * *p* < 0.05 CTRL vs. DBK + DOX; *** *p* < 0.05 CTRL vs. TMZ 500 µM, TMZ 750 µM and TMZ 1000 µM; ^•^ *p* < 0.05 CTRL vs. DBK + TMZ 500 µM, DBK + TMZ 750 µM and DBK + TMZ 1000 µM; ^••^ *p* < 0.05 CTRL vs. DOX + TMZ 500 µM, DOX + TMZ 750 µM and DOX + TMZ 1000 µM; ^•••^ *p* < 0.05 CTRL vs. DBK + DOX + TMZ 500 µM, DBK + DOX + TMZ 750 µM and DBK + Dc’OX + TMZ 1000 µM. (**B**) * *p* < 0.05 CTRL vs. DBK + DOX; ** *p* < 0.05 CTRL vs. DOX; *** *p* < 0.05 CTRL vs. TMZ 500 µM, TMZ 750 µM and TMZ 1000 µM; ^•^ *p* < 0.05 CTRL vs. DBK + TMZ 500 µM and DBK + TMZ 1000 µM; ^••^ *p* < 0.05 CTRL vs. DOX + TMZ 1000 µM; ^•••^ *p* < 0.05 CTRL vs. DBK + DOX + TMZ 750 µM and DBK + DOX + TMZ 1000 µM. (**C**) * *p* < 0.05 CTRL vs. DBK + DOX, *** *p* < 0.05 CTRL vs. TMZ 500 µM, TMZ 750 µM and TMZ 1000 µM; ^••^ *p* < 0.05 CTRL vs. DOX + TMZ 500 µM, and DOX + TMZ 1000 µM; ^•••^ *p* < 0.05 CTRL vs. DBK + DOX + TMZ 500 µM, DBK + DOX + TMZ 750 µM and DBK + DOX + TMZ 1000 µM. ANOVA (Holm–Sidak test). Number of experiments = 4.

Considering the effects of kinin agonists on glioblastoma cells [27], we examined whether the transient opening of the BBB by 1 µM DBK could inadvertently impact tumor suppression if a portion of the peptide crosses into the brain parenchyma. Cell viability assays in glioblastoma cell lines (T98G, U87MG, and C6) demonstrated that DBK alone does not alter cell viability (Figure 9). However, when combined with DOX, DBK significantly reduced the viability of all tested cell lines. Additionally, the combination of DBK, DOX, and TMZ decreased cell viability in U87MG cells at TMZ concentrations of 750 µM and 1000 µM (Figure 9A), while in T98G and C6 cells, this effect was observed at 500 µM TMZ (Figure 9B,C).

## 3. Discussion

Several studies emphasize the importance of optimizing and identifying therapeutic targets that can bypass the BBB to enhance drug efficacy in the CNS. Combining strategies aimed at improving the penetration of antitumor and pharmacological agents, which transiently modulate the barrier, may result in better treatment outcomes.

Brain capillary endothelial cells are interconnected by tight junctions [62]. Analysis of ZO-1, a key protein in the blood-brain barrier (BBB), revealed that CMT98G increased ZO-1 staining and mRNA levels. However, this may not correlate with the formation of functional tight junctions. Both control cells (cultured in DMEM/F-12) and those treated with CMT98G exhibited reduced ZO-1 staining intensity upon the addition of 1 µM DBK, suggesting modification of the blood-brain barrier. Activation of the B1R by DBK alters intracellular Ca^2+^ levels, promoting actin reorganization [63], which may impact endothelial junction proteins, such as ZO-1. This reduction in ZO-1 staining could indicate either the disorganization of endothelial junctions or a protective rearrangement of the BBB to shield against harmful substances or pharmacological agents targeting the brain parenchyma.

A non-invasive experimental strategy to modulate BBB opening involves the systemic injection of vasoactive agents, such as kinins [43]. B1R overexpression has been reported in murine and human glioma cell lines, including LN229, T98G, U118, U138, and U87MG, reflecting the levels observed in glioma patients but not in normal brain tissue [43]. Exposure of endothelial cells to conditioned media from T98G and U87MG glioblastoma cell lines did not alter B1R expression patterns, even with the addition of 1 µM DBK.

Considering the potential relationship between B1R and TLR4 [53], proximity ligation assays between B1R and TLR4 revealed a decrease in proximity points upon the addition DBK to conditioned media. This suggests that variations in B1R or TLR4 expression, whether upregulated or downregulated, reduce the interaction between these receptors. Gene expression analysis revealed an increase in B1R mRNA in HBMECs treated with CMT98G and CMU87, with levels returning to baseline upon DBK addition. In contrast, TLR4 mRNA levels remained unchanged under conditions of increased protein expression (CMT98G + DBK and CMU87 + DBK). Immunocytochemistry images revealed that B1R and TLR4 were localized in the perinuclear region of cells exposed to conditioned media, both with and without DBK, as well as in control situations. In the U87MG cell line, high B1R expression was observed, potentially increasing further with DBK administration [64]. Pro-inflammatory factors in the tumor microenvironment play a crucial role in upregulating B1R during tumor invasion, a process characterized by chronic inflammation [13,64]. Glioblastoma cell lines exhibit distinct molecular profiles in their secretomes, which influence tumor migration and growth [65]. For instance, U87MG cell line is sensitive to the chemotherapy drug TMZ, whereas T98G is resistant [66]. These differences may lead to variations in the factors released into the culture media, which could explain why CMU87 had no effect on endothelial cell growth or morphology, while CMT98G induced morphological changes in HBMECs.

In the glioblastoma cell lines, BK binds to B1R, activating signaling pathways associated with proliferation and migration. This raises concerns regarding the use of B2R agonists for BBB modulation [67]. Notably, capillary endothelial cells in both normal brain tissue and brain tumors do not express kinin B_2_ receptors. However, their presence in tumor cells facilitates barrier permeability [68]. B2R is considered the primary mediator of kinin effects under physiological conditions and during acute inflammation, whereas B1R is mainly involved in chronic inflammatory responses [69,70]. This aligns with mRNA analysis data, which showed significant increases in B1R expression in HBMECs treated with T98G- and U87MG-conditioned media for 24 h. Additionally, DBK addition maintained elevated B1R levels in CMT98G-treated cells.

The transcription factor NF-κB, which regulates gene expression during inflammation, is a key mediator of B1R induction [69,70,71]. Stimulation by agents, such as LPS, triggers a signaling cascade that translocates active NF-κB to the nucleus, where it modulates the transcription of pro-inflammatory mediators [71,72]. NF-κB is also associated with cell proliferation, transformation, and tumor development [73], highlighting the role of kinins in tumor development and progression [69]. In U87MG cells, NF-κB levels increased following exposure to BK [74]. However, these cytokine-driven effects were not observed in endothelial cells treated with conditioned media from T98G and U87MG. While CMT98G and CMU87, with or without DBK, did not stimulate NF-κB gene expression, the protein may still be active, translocating to the nucleus to regulate the transcription of inflammatory genes.

Tight junctions, molecular adhesions, and ion channels regulate the influx and efflux of ions, such as Ca^2+^ and K^+^ [20]. The modulation of K_Ca_ through B2R activation or blockade enhances BBB permeability [40], and K_Ca_ and K_ATP_ influence endothelial cell responses to DBK [53]. An increase in intracellular Ca^2+^ levels could mediate the cellular response of B1R to DBK, triggering conformational changes in BBB-associated proteins, such as ZO-1.

The reduction in ZO-1 staining intensity following the incubation of HBMECs with 1 µM DBK, even after the high intensity observed in CMT98G-treated cells, suggests BBB opening. However, this opening must be transient and reversible to effectively enhance drug bioavailability to the brain parenchyma, making it a potential adjuvant therapy. This apparent decrease in ZO-1 may result in the disorganization of endothelial junctions and the BBB, or it could reflect a rearrangement that protects the BBB from the passage of harmful substances into the brain parenchyma or even from pharmacological molecules. Wang and colleagues (2020) [75] indicated in an ischemia model that there was a reduction in claudin-5 expression, but this did not increase the permeability of the BBB. In MCT998G, there was also an increase in claudin-5 mRNA, a transmembrane protein that can regulate the paracellular permeability of small molecules [62], which appears not to be negatively regulated in most tumor microvessels [76].

The clinical development of RMP-7, a BK analog, aimed at opening the BBB, failed in phase II trials due to insufficient clinical benefits [30]. One possible explanation is the relatively short duration of barrier opening, which did not significantly enhance the chemotherapeutic availability of the chemotherapeutic agent within tumors [30]. Beyond B2R involvement, LPS-induced responses highlight the roles of both TLR4 and B1R in BBB modulation. However, systemic LPS doses must be carefully controlled to trigger the release of the pro-inflammatory mediator without causing septic shock [77,78]. Systemic LPS administration does not induce ultrastructural changes in cerebral vessels significant enough to alter their permeability, although tight junction integrity was not assessed [77]. The mechanism of selective barrier opening remains unclear, but evidence suggests that kinin receptor manipulation increases Ca^2+^ influx into endothelial cells, potentially destabilizing tight junctions [45,53,79].

The 24 h BBB opening observed in vitro was confirmed in vivo. At 24 h post-DBK injection, a significant presence of Evans blue was detected in the brain parenchyma of both control animals and those inoculated with C6 cells. By 48 h, the amount of dye was comparable to that in control animals, with or without C6 inoculation, which had not received DBK injections. In EB extravasation assays, BK and a pharmacological analog selectively increased BBB permeability in glioma-bearing rats, peaking 15 min after B2R agonist infusion [80,81], corroborating our results.

In vitro temporal analysis of B1R mRNA expression in portal vein endothelial cells showed detectable levels as early as 30 min after DBK incubation, peaking at 6 h and persisting for at least 12 h [14]. Interestingly, tumor-induced barrier disruption did not enhance EB passage compared to the control group. Glioblastoma’s abnormal vasculature, driven by upregulated angiogenic factors, led to the formation of new blood vessels within the tumor. However, this vascular network exhibits increased permeability that does not translate into enhanced drug bioavailability to the tumor mass [57].

Sikpa and colleagues (2020) [45] demonstrated that simultaneous injection of B1R and B2R agonists, along with the chemotherapeutic agent DOX, enhanced BBB opening and drug bioavailability in brain tissue. Our findings align with this but focus on DBK, which primarily activates B1R and suggest that DBK would only bind to B2R in the complete absence of B1R [53]. Furthermore, qualitative analysis revealed a greater presence of DOX in the brain tumor region in histological sections from animals treated with DBK compared to those that received DOX alone. However, this was not corroborated when DOX was extracted from the brain parenchyma and quantified. This discrepancy may be attributed to the low number of experimental replicates per condition and the variability in tumor size among animals, as C6 is a rat-derived cell line injected into mice. In both EB and DOX quantification experiments, tumor size significantly influenced the amount of agent incorporated. Notably, tumors in animals receiving only DOX may have been larger than those in animals pretreated with DBK before DOX administration.

**Figure 10:**
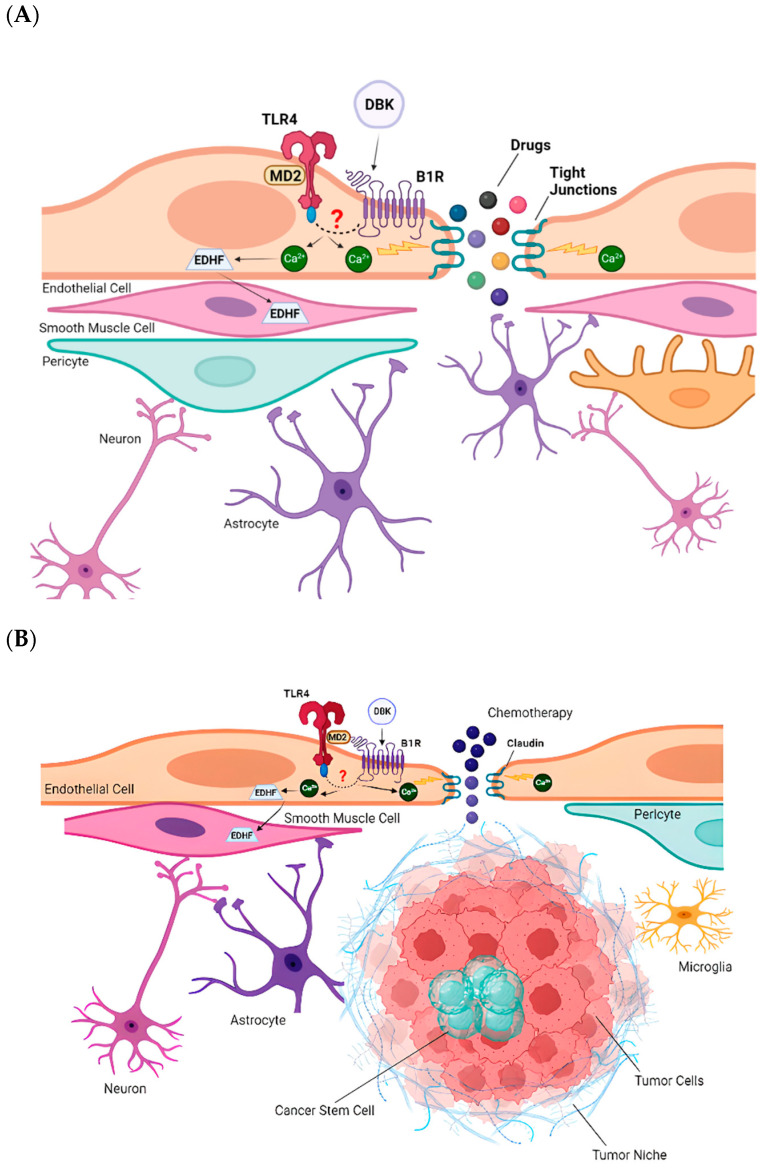
Hypothesis for DBK-mediated transient BBB opening. (**A**) Activation of B1R by DBK promotes the interaction with TLR4, leading to an increase in intracellular calcium concentration. This destabilizes the tight junctions that form the barrier between endothelial cells in blood vessels, resulting in enhanced drug flow into the brain parenchyma. Concurrently, elevated calcium levels stimulate the production of EDHF, which is transported to smooth muscle cells, inducing hyperpolarization. (**B**) DBK-mediated activation of the B1R–TLR4 interaction increases intracellular calcium, disrupting key blood-brain barrier proteins. This disruption may enhance BBB permeability, allowing higher concentrations of chemotherapeutic agents to reach the tumor mass. In addition to DBK, another potential adjuvant at very low doses is LPS, which could induce the differentiation of tumor stem cells, thereby enhancing the efficacy of chemotherapeutic agents, such as TMZ.

Our data suggest that DBK may stimulate cells and permeability not only through B1R activation, but also via B2R. Collectively, the data presented here, along with evidence from the literature, support the hypothesis that B1R activation by DBK promotes interaction between B1R and TLR4, as indicated in the group’s previous study [53]. This interaction increases intracellular calcium concentration, destabilizing the tight junctions that form barriers between endothelial cells in blood vessels (Figure 10A). Consequently, drug flow into the brain parenchyma is enhanced. Meanwhile, the increase in intracellular calcium concentration elevates the levels of endothelium-derived hyperpolarizing factor (EDHF), which is transported to the smooth muscle, where it induces hyperpolarization [52,53].

In inflammatory contexts, local B1R and TLR4 expression [56] supports the hypothesis that DBK enhances drug bioavailability to the tumor mass. Activation of TLR4 and B1R increases intracellular calcium levels, disrupting proteins that compose the BBB, such as ZO-1 (Figure 10B).

## 4. Materials and Methods

### 4.1. Animals

Experiments were carried using both male and female Swiss mice. The animals were 10 weeks old, with a weight range of 20–30 g. The mice were provided by the rodent facility of the Instituto de Ciências Biomédicas (ICB) at Universidade Federal do Rio de Janeiro (UFRJ), Brazil. They were housed in groups of three to four per cage and maintained under controlled conditions of temperature, humidity, and a 12:12 h light–dark cycle, with chow pellets and tap water available ad libitum.

All procedures were approved by the Ethics Committee on the Use of Animals in Research of the Universidade Federal do Rio de Janeiro (protocol number: 023/19 and 134/22).

### 4.2. Maintenance of Cell Lines

For in vitro experiments, cell cultures were established using HBMEC, human glioblastoma cell lines (U87MG and T98G), and the rat glioma cell line C6.

HBMECs were provided by Dr. Ana Paula Cabral de Araújo Lima (Instituto de Biofísica Carlos Chagas Filho, UFRJ, Rio de Janeiro, Brazil) with the consent of Dr. Dennis Grab (Associate Professor, Johns Hopkins University, Department of Pathology, Baltimore, MD, USA). HBMECs were plated or not onto glass coverslips in a 24-well plate at a density of 4 × 10^4^ per well and 4 × 10^5^ in 25 cm^2^ cell culture flasks. Cells were maintained in medium 199 (M199; 31100035, Gibco/ThermoFisher Scientific, Waltham, MA, USA) supplemented with 1% glutamine-penicillin-streptomycin (100X) (50 mg/mL) (10378016, Gibco/ThermoFisher Scientific, USA), and 10% fetal bovine serum (FBS; 12657029, Gibco/ThermoFisher Scientific, USA) for 5–7 days until confluence and used from passage 35–40.

U87MG and T98G cells were cultured in DMEM/F-12 medium (11320082, Gibco/ThermoFisher Scientific, USA), supplemented with 10% FBS and 1% penicillin/streptomycin/glutamine. Cells were plated on 35 × 10 mm dishes. After 24 h in contact with the culture, the media (conditioned media CMU87 or CMT98G) were removed, filtered (0.22 µm filter), and stored frozen at −80 °C.

The C6 cell line was provided by Dr. Patrícia Pestana Garcez (ICB/UFRJ) and cultured in 25 cm^2^ cell culture flasks in DMEM/F-12 medium supplemented with 10% FBS and 1% penicillin/streptomycin/glutamine. After 80–90% confluence (5–7 days), cells were detached using 3 mL of trypsin/EDTA (0.5%) (15400054, Gibco/ThermoFisher Scientific, USA) and resuspended in 1 mL of DMEM/F-12 with FBS. After counting in a Neubauer chamber, cells were adjusted to a concentration of 3 × 10^5^ cells per µL for the injection of a 3 µL volume into the brain parenchyma of Swiss mice.

HBMECs were cultured in medium 199 until they reached 100% confluence, after which the cells were exposed for 24 h to the following different conditions: DMEM/F-12 FBS free, DMEM/F-12 with FBS, 199 FBS free, 199 with FBS, astrocyte-conditioned medium (ACM), CMU87, and CMT98G. After 24 h, images of the endothelial cells were captured on an EVOS M5000 microscope (ThermoFisher Scientific, USA) and subsequent analysis of cell density and morphology was conducted. These cells were also incubated for 24 h in DMEM/F-12 with FBS, CMU87, or CMT98G media, with or without 1 µM DBK, provided by Dr. João Bosco Pesquero (Departamento de Biofísica, Unifesp, Sao Paulo, Brazil). Cells plated on coverslips were fixed with 4% PFA for 20 min and processed for immunofluorescence or proximity ligation assay protocols. Cells plated without coverslips were incubated with TRIzol^TM^ reagent (15596026, Invitrogen/ThermoFisher Scientific, USA) for RNA extraction protocol.

### 4.3. mRNA Expression

Total RNA was isolated using TRIzol^TM^. One µg of total RNA from thoracic aorta was reverse transcribed using a high-capacity kit (4368814, Applied Biosystems™/ThermoFisher Scientific, USA) for cDNA, as suggested by the manufacturer. The reaction product was amplified by real-time PCR (QuantStudio^TM^ 7 Flex Real-Time PCR System, Applied Biosystems™/ThermoFisher Scientific, USA) using 1:10 diluted cDNA and primers (600 nM) for fast SyberGreen reaction (A25742, Applied Biosystems™/ThermoFisher Scientific, USA). For data analysis, the quantitative values for the expression were obtained from the 2^−ddCt^ parameter, in which dCt represents the subtraction of the Ct values of the reference gene from those of the target gene and ddCt the normalization of the samples to the sample group mean. The final data are expressed in the ratio of the relative expression (fold-change) of the alteration in the target gene of the treated experimental groups over the variation in the target gene of the endothelial cells of the control experimental groups. For better analysis and visualization of fold changes in the data obtained, the graphs will be presented using the log base 2. The primers used for real-time PCR (Exxtend Biotechnologia, Sao Paulo, Brazil) were as follow: human/mouse GAPDH forward primer 5′-CCCATCACCATCTTCCAGGA-3′ and reverse primer 5′-ATGATGACCCTTTTGGCTCC-3′; human/mouse B1R forward primer 5′-TGCCCTTCTGGGCAGAGAA-3′ and reverse primer 5′-CTGATGGCCACCACCAGGAA-3′; human/mouse B2R forward primer 5′-CATGAACCTGTACAGCAGCA-3′ and reverse primer 5′-GTACAGCCCCAGATCACC-3′; human/mouse TLR4 forward primer 5′-GCTGGATTTATCCAGGTGTG-3′ and reverse primer 5′-TTTGTCTCCACAGCCACC-3′; human ZO-1 forward primer 5′-ACCAGTAAGTCGTCCTGATCC-3′ and reverse primer 5′-TCGGCCAAATCTTCTCACTCC-3′; human Claudin-5 forward primer 5′-TCTCTCCTGTCTGAAGGCCA-3′ and reverse primer 5′-GGCTTCCCAGACCTCTCAATC-3′; human/mouse NF-kB forward primer 5′-CTTCCGGCTGAGTCCTGCTC-3′ and reverse primer 5′-AGGCTGCCTGGATCACTTCA-3′.

### 4.4. Immunocytochemistry and Immunohistochemistry

Cells cultured in glass coverslips were fixed in 4% PFA for 20 min and was permeabilized with 0.2% Triton^TM^ X-100 (X-100, Sigma-Aldrich/Merck, St Louis, MO, USA) diluted in phosphate-buffered saline 1x (PBS; 10010023, Gibco/ThermoFisher Scientific, USA), for 5 min at room temperature, and nonspecific sites were blocked with 10% bovine serum albumin (BSA; A7906, Sigma-Aldrich/Merck, USA) and normal goat serum 5% (NGS; 16210064, Gibco/ThermoFisher Scientific, USA) for 2 h before immunoreactions with the following antibodies, diluted in blocked solution: rabbit anti-ZO-1 (61-7300, Invitrogen/ThermoFisher Scientific, USA) (dilution 1:100), rabbit anti-BDKRB1 (BS-8675R, Bioss/ThermoFisher Scientific, USA) (dilution 1:300), and mouse anti-TLR4 (MA5-16216, Invitrogen/ThermoFisher Scientific, USA) (dilution 1:300). After 12–16 h of primary antibody incubation, cells were thoroughly washed with PBS and incubated with secondary antibodies, diluted in BSA 1% for 2 h at room temperature. Secondary conjugated antibodies were as follows: goat anti-mouse IgG Alexa Fluor^TM^ 546 (A-11030, Invitrogen/ThermoFisher Scientific, USA) (dilution 1:1000), goat anti-rabbit IgG Alexa Fluor^TM^ 546 (A-11035, Invitrogen/ThermoFisher Scientific, USA) (dilution 1:1000), goat anti-rabbit IgG Alexa Fluor^TM^ 488 (A-11034, Invitrogen/ThermoFisher Scientific, USA) (dilution 1:1000), and goat anti-mouse IgG Alexa Fluor^TM^ 488 (A-11029, Invitrogen/ThermoFisher Scientific, USA) (dilution 1:1000). Nuclei were counterstained with 4′,6-Diamidino-2-phenylindole (DAPI; D1306, Invitrogen/ThermoFisher Scientific, USA) for 5 min. Glass coverslips or aorta slices were mounted in glass microscopy slides with Fluoromount-G^TM^ mounting medium (00-4958-02, Invitrogen/ThermoFisher Scientific, USA) and visualized using a Leica TCS SP5 confocal microscope (Leica Microsystems, Wetzlar, Germany).

After incubation with ZO-1 antibody, cells were washed three times with PBS and incubated with phalloidin-FITC (P5282, Sigma-Aldrich/Merck, USA) for F-actin cytoskeleton staining, for 1.5 h at room temperature in a dark and humid chamber.

The specificity of the antibody for B1R was tested in HEK293T cells as a negative control for B1R [53].

### 4.5. In-Cell Western Assay

As described by Batista et al. (2024) [53], protein levels of B1R and TLR4 were determined using the in-cell Western assay. HBMECs fixed with 4% PFA were then permeabilized with 0.1% Triton^TM^ X-100 diluted in PBS, for 5 min at room temperature. Nonspecific antibody binding sites were blocked with blocking buffer (5% NGS, 0.3% Triton^TM^ X-100 diluted in PBS) for 2 h at room temperature. Cells were incubated with primary antibody in antibody-diluting buffer (1% BSA, 0.3% Triton^TM^ X-100 diluted in PBS) overnight at 4 °C. The next day, cells were washed three times with 0.1% Triton^TM^ X-100 diluted in PBS for 5 min at room temperature. The appropriate LI-COR IRDye^®^ secondary antibodies were diluted (1:1000) in an antibody-diluting buffer, and the cells were incubated in darkness for 1 h at room temperature. The primary antibodies used were rabbit anti-BDKRB1 (dilution 1:300), mouse anti-TLR4 (dilution 1:300), and rabbit anti-ß-actin (ab8227, Abcam, UK) (dilution 1:500). The secondary antibodies used were goat anti-mouse IRDye^®^800 CW (926-32210, LI-COR Biotech, Lincoln, NE, USA) (dilution 1:800) and goat anti-mouse IRDye^®^680 LT (926-68050, LI-COR Biotech, USA) (dilution 1:800). Cells were then washed three times with 0.1% Triton^TM^ X-100 diluted in PBS. The LI-COR Odyssey (LI-COR Biotech, USA) glass scanner surface was wiped with water and the plate was scanned using both 700 and 800 nm channels. Data were analyzed using the Un-Scan-It gel software, version 6.1 (Silk Scientific, Inc., Vineyard, UT, USA). The images were converted to black and white, and the grayscale of each well’s image was analyzed, with density calculated in linear mode. Densities for each condition were determined by normalizing the target marker value to β-actin.

### 4.6. Proximity Ligation Assay

As described by Batista et al. (2024) [53], after fixation with 4% PFA for 20 min, the HBMECs cultured in glass coverslips were processed as previously described for the Duolink^®^ PLA In Situ Fluorescence mouse/rabbit Kit (DUO92101, Sigma-Aldrich/Merck, USA). Cells were washed for 5 min, three times, with PBS, and were incubated in a humidified chamber with Duolink^®^ Blocking Solution for 1 h at 37 °C; then, the blocking solution was removed and replaced with the primary antibodies diluted in Duolink^®^ Antibody Diluent overnight at room temperature. The primary antibodies used for PLA were rabbit anti-BDKRB1 (dilution 1:300) and mouse anti-TLR4 (dilution 1:300). The coverslips were washed twice for 5 min with Wash Buffer A, and then the Duolink^®^ In Situ PLA Probe Anti-Rabbit PLUS and Duolink^®^ In Situ PLA Probe Anti-Mouse MINUS PLA probes (Sigma-Aldrich/Merck, USA) diluted in Duolink^®^ Antibody Diluent were applied. The coverslips were incubated in a humidified chamber with PLA probe solution for 1 h at 37 °C. Coverslips were washed twice with Wash Buffer A, followed by the addition of the ligation solution for 30 min at 37 °C, then another two washing steps, and incubation in amplification solution for 100 min at 37 °C. The coverslips were washed twice for 10 min in Wash Buffer B and mounted onto slides with Duolink^®^ In Situ Mounting Medium with DAPI.

### 4.7. Paracellular Permeability Assay

HBMECs were seeded (5 × 10^4^ cells) onto cell culture inserts with a pore size of 0.4 µm (140620, Thermo Scientific™/ThermoFisher Scientific, USA). Once 100% confluence was achieved, 400 µL of DMEM/F-12 with FBS or conditioned media from T98G (CMT98G) or U87MG (CMU87) cells were added to the basal chamber of the culture inserts. To the apical side of the confluent monolayer, 200 µL of DMEM/F-12 with FBS with or without 1 µM DBK was added.

Twenty-three hours after DBK administration, 200 µL of 1% EB was added to the apical chamber of the insert. One hour later (twenty-four hours after DBK administration), the medium from the basal chamber was collected and plated in a 96-well plate (50 µL/well) in triplicate. The presence of the dye was measured at an absorbance of 620 nm using a FlexStation 3 plate reader (Molecular Devices, Silicon Valley, CA, USA) with the SoftMax Pro 5.4 software (Molecular Devices, USA). The dye concentration in the basal medium was calculated based on a standard curve (mg/mL) with reference points ranging from 0 to 10 mg/mL.

### 4.8. Evaluation of BBB Integrity Using Evans Blue

In vivo experiments were conducted using Swiss mice (total number of animals = 72), which were divided into the following two groups: the control group (CTRL) and the C6 group, with mice inoculated with C6 cell line to induce tumor formation in the brain parenchyma.

For the surgical procedure, each animal was anesthetized using a combination of a muscle relaxant and anesthetic, specifically ketamine (Syntec, Sao Paulo, Brazil) and xylazine (Syntec, BR), administered intraperitoneally at doses of 100 mg/kg and 20 mg/kg, respectively. Using predetermined stereotaxic coordinates for injection into the caudate putamen (coordinates relative to the bregma: 0.5 mm posterior, 2.0 mm left, and 3.5 mm depth), 5 × 10^5^ C6 cells diluted in 3 μL of serum-free DMEM/F-12 were injected. The CTRL animals underwent the same surgical procedure but received 3 μL of cell-free DMEM/F-12.

In the first experiment, to analyze the transient opening of the BBB, control animals and those inoculated with C6 cells were treated with 1 µM DBK (diluted in 100 µL of saline for subcutaneous injection in the groin region) [80,81]. Twenty-three or forty-seven hours after the DBK injection, the animals received an injection of 2% Evans blue solution (100 µL) in the tail vein [82].

One hour after the EB injection, the animals were euthanized with a lethal dose of ketamine and xylazine at three times the concentration required for anesthesia. They were then subjected to transcardiac perfusion with 0.9% saline using a peristaltic pump for 10 min. After perfusion, the brains were removed, weighed, and immersed in 500 µL of formamide (F9037, Sigma-Aldrich/Merck, USA) for 48 h at room temperature to extract the dye present in the brain tissue.

The formamide from each sample was plated in 96-well plates (triplicates for each sample) at a volume of 100 µL per well. The samples were measured at an absorbance of 620 nm [83,84]. A blank well containing 100 µL of pure formamide was used as the control for the reading. Dye concentration was calculated as the absorbance ratio relative to the tissue weight (OD620 nm/g tissue) [83,84].

### 4.9. Doxorubicin Delivery in Mouse Brain

Control mice and those inoculated with C6 cells were treated with 4 mg/kg DOX (100 µL, tail vein injection) [45], with or without 1 µM DBK (100 µL, subcutaneous injection in the groin region). DOX (44583, Sigma-Aldrich/Merck, USA) was administered 1 h after DBK injection. Then, 24 h following peptide injection, mice were euthanized and subjected to transcardiac perfusion. Brains were removed and separated into two groups, with one designated for histological sections and the other for the extraction of DOX present in the brain parenchyma.

Brain tissue samples designated for histological analysis were immersed in 4% PFA at room temperature. After 24 h, the samples were cryopreserved sequentially in 10%, 20%, and 30% sucrose solutions, remaining for approximately 24 h in each. Finally, the tissue was embedded in Tissue-Tek O.C.T. mounting medium for cryostat sectioning (Sakura Finetek, Torrance, CA, USA). To observe the fluorescence properties of DOX, nuclei were stained with DAPI, and the slides were mounted Fluoromount-G. Images were captured using confocal microscopy.

For brain samples designated for DOX extraction, tissues were weighed and immersed in 500 µL of acidified ethanol (50% ethanol in 0.3 N HCl). The samples were minced and incubated at 4 °C for 24 h and then centrifuged at 16,000× *g* for 25 min, and the supernatant was collected. The samples were plated in 96-well plates (triplicates for each sample) at a volume of 100 µL per well. DOX concentration in the diluent was measured at an absorbance of 496 nm [45]. The concentration of DOX was calculated as the absorbance ratio relative to the tissue weight (OD496 nm/g tissue).

### 4.10. Histological Staining with Crystal Violet or Hematoxylin and Eosin (H&E)

Brain tissue sections (6 µm thick) from control animals and those inoculated with C6 cells were stained using crystal violet, to delimit the tumor region from the normal tissue, or H&E staining, to characterize the tumor mass at the cellular level.

For crystal violet staining, the following protocol was used: water for 5 min, crystal violet solution for 5 h, water for 15 min, and 10 µL of Entellan^TM^ resin (100869, Sigma-Aldrich/Merck, USA) after the sections were dry.

The crystal violet stock solution was prepared using 14 g of crystal violet and 100 mL of 95% ethanol. The working solution was prepared using 10 mL of crystal violet stock solution, 300 mL of distilled water, and 1 mL of concentrated hydrochloric acid.

For H&E staining of brain sections, the following steps were performed: water for 5 min, hematoxylin for 10 min, water for 10 min, eosin for 2 min, water for 5 min, 70% ethanol for 5 min, 80% ethanol for 5 min, 90% ethanol for 5 min, 100% ethanol for 5 min, xylene I for 5 min, and xylene II for 5 min; we then added 50 µL of Entellan^TM^ and placed a coverslip on the slide.

Images were captured using an Axio Imager A.1 (Zeiss, Oberkochen, Germany) light microscope with 20x or 40x objective lenses.

### 4.11. Cell Viability Analysis Using MTT Assay

The cytotoxic effect of 1 µM DBK on the tumoral cell lines (T98G, U87MG, and C6) was evaluated using the MTT assay (3-(4,5-Dimethylthiazol-2-yl)-2,5-Diphenyltetrazolium Bromide; M6494, Invitrogen/ThermoFisher Scientific, USA). Cell lines were seeded in 96-well plates (2 × 10^4^ cells per well) and maintained in 100 µL of DMEM/F-12. After 24 h, cells were treated with a final volume of 100 µL DMEM/F-12 containing the following treatment conditions: control, DBK 1 µM, DOX 5 µM [85], DBK + DOX, TMZ 500 µM [66], TMZ 750 µM, TMZ 1000 µM, TMZ 500 µM + DOX, TMZ 750 µM + DOX, TMZ 1000 µM + DOX, TMZ 500 µM + DOX + DBK, TMZ 750 µM + DOX + DBK, and TMZ 1000 µM + DOX + DBK.

After 72 h, 0.5 mg/mL of MTT solution was added to each well, the plates were incubated for 2 h, and then 100 µL of DMSO was added to the cells and the absorbance (570 nm) was measured using a plate reader. Cell viability was calculated using a standard curve with different MTT concentrations in dimethyl sulfoxide (DMSO; D2438, Sigma-Aldrich/Merck, USA).

### 4.12. Statistical Analysis

GraphPad software, version 8.0 (GraphPad Software Inc., La Jolla, CA, USA), was used for statistical analysis of the quantitative data. The analysis of variance test was applied in all comparisons, followed by Tukey, Holm–Sidak, Mann–Whitney, or Kruskal–Wallis tests. Data are reported as mean ± S.E., and error bars in the graphs represent S.E.

## 5. Conclusions

The discovery of mechanisms to enhance drug delivery to brain tissue is a research avenue that has been explored by numerous groups. Our data support findings from other studies, suggesting a more physiological approach, namely the use of the DBK peptide, which focuses on activating B1R rather than B2R or both, as proposed in other studies for the transient barrier opening. The interaction between B1R and TLR4 in endothelial cells presents a promising target for transiently opening the BBB. The administration of DBK induces BBB opening for less than 48 h in both the cerebral environment with and without glioblastoma. The peptide’s action facilitates the passage of DOX and, in the presence of glioblastoma, it appears to concentrate in the tumor mass region. We suggest that DBK acts particularly on vessels in regions experiencing inflammation, where there is likely a higher concentration of B1R. Complementary studies will be necessary to further investigate B2R activity in this model and the effect of the agonist on the other proteins constituting the barrier.

## Data Availability

All data that support the findings and conclusions of this study are included within the article.

## Supplementary Materials

The following supporting information can be downloaded at: https://www.mdpi.com/article/10.3390/ph18040591/s1, Figure S1: CMT98G alters the mRNA expression of claudin-5 in endothelial cells, but this effect is not observed when DBK is added to the medium; Figure S2: Relative quantification of B1R and TLR4 staining in HBMEC cells was performed using the In-Cell Western assay; Figure S3: The relative levels of B2R mRNA expression in endothelial cells incubated for 24 h with conditioned media from MCT98G or MCU87, with or without DBK; Figure S4: Histological staining with crystal violet. Brain tissue sections from control mice (A) and mice 20 days post-inoculation with C6 cells (B)**.**

## Abbreviations

The following abbreviations are used in this manuscript:

ACM	Astrocyte-conditioned medium
BBB	Blood-brain barrier
BK	Bradykinin
B1R	Kinin B_1_ receptor
B2R	Kinin B_2_ receptor
CMT98G	Conditioned media from glioblastoma cell line T98G
CMU87	Conditioned media from glioblastoma cell line U87MG
CNS	Central nervous system
CTRL	In vivo experimental control group
C6	Glial cell line isolated from the brain of rat with glioma
DAPI	Nuclear staining 4′,6-Diamidino-2-phenylindole
DBK	des-Arg^9^-bradykinin
DMEM/F-12	Dulbecco’s modified Eagle’s medium F12
DMSO	Dimethyl sulfoxide
DOX	Doxorubicin
EB	Evans blue dye
EDHF	Endothelium-derived hyperpolarizing Factor
FBS	Fetal bovine serum
HBMECs	Human brain microvascular endothelial cells
LPS	Lipopolysaccharide
Lys-BK	Lys-bradykinin
Lys-DBK	Lys-des-Arg^9^-bradykinin
mRNA	Messenger ribonucleic acid
NF-κB	Nuclear factor kappa B
PFA	Paraformaldehyde
PLA	Proximity ligation assay
RT-qPCR	Reverse transcription quantitative real-time polymerase chain reaction
TLR4	Toll-like Receptor 4
TMZ	Temozolomide
T98G	Human cell line derived from glioblastoma tumor
U87MG	Human cell line derived from glioblastoma tumor
