# Supplementary material for: Kinin B1 Receptor Agonist Enhances Blood-Brain Barrier Permeability in Healthy and Glioblastoma Environments
Source: Pharmaceuticals (Basel). 2025 Apr 18;18(4):591. doi: 10.3390/ph18040591 (PMC12030169; doi:10.3390/ph18040591)
Supplement: Supplementary file 1 [file pharmaceuticals-18-00591-s001.zip › pharmaceuticals-3529534-supplementary.pdf]

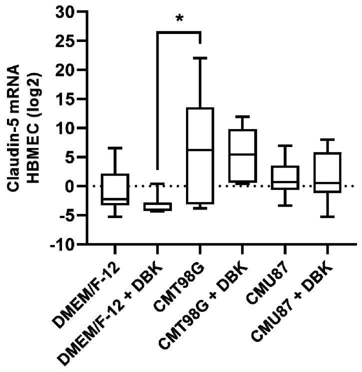

Fig. S1 CMT98G alters the mRNA expression of claudin-5 in endothelial cells, but this effect is not observed when DBK is added to the medium. Specifically, claudin-5 mRNA expression is increased in HBMEC cells incubated with MCT98G but decreases when DBK is present in the medium. *p<0.05 MCT98G vs DMEM/F-12 + DBK (Kruskal-Wallis Test). Number of experiments = 7.

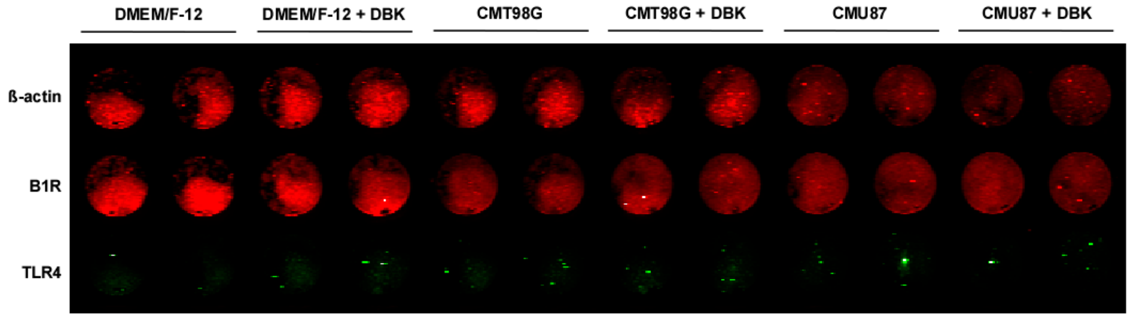

Fig. S2 Relative quantification of B1R and TLR4 staining in HBMEC cells was performed using the In-Cell Western assay. Representative images of the assay show staining for B1R, TLR4, and β-actin. Number of experiments = 7.

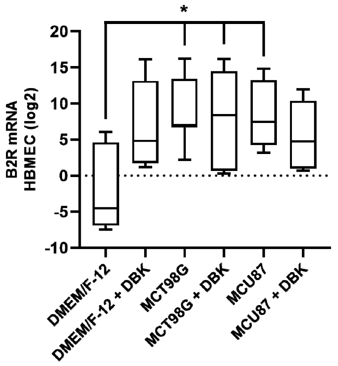

Fig. S3 The relative levels of B2R mRNA expression in endothelial cells incubated for 24 hours with conditioned media from MCT98G or MCU87, with or without DBK. The analysis of B2R expression in HBMEC cells revealed an increase in B2R mRNA levels when exposed to conditioned media from MCT98G cells, with an even greater upregulation observed when DBK was added to the medium. *p<0.05 DMEM/F-12 vs MCT98G, MCT98G + DBK e MCU87 (Kruskal-Wallis Test). Number of experiments = 9.

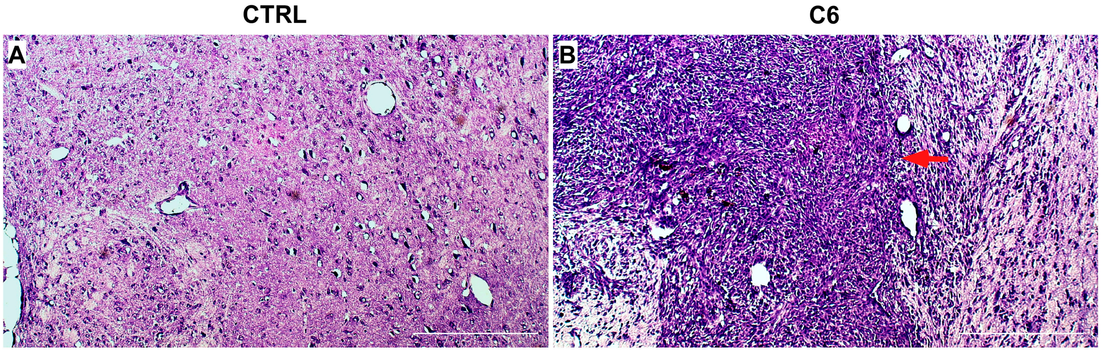

Fig. S4 Histological staining with crystal violet. Brain tissue sections from control mice (A) and mice 20 days post-inoculation with C6 cells (B). The red arrow in **(B)** indicates the peritumoral region, characterized by a darker staining, which reflects the high cellular density in this area. Light microscopy; scale bar: 250 μm.
